# Editorial: Recent Advances in Ovarian Stimulation

**DOI:** 10.3389/fendo.2022.874628

**Published:** 2022-04-08

**Authors:** Panagiotis Drakopoulos, Federica Di Guardo, Nicola Pluchino, Antonis Makrigiannakis

**Affiliations:** ^1^ Centre for Reproductive Medicine, Universitair Ziekenhuis Brussel, Vrije Universiteit Brussel, Brussels, Belgium; ^2^ Department of Obstetrics and Gynaecology, University of Alexandria, Alexandria, Egypt; ^3^ In Vitro Fertilisation (IVF) Athens, Athens, Greece; ^4^ Department of General Surgery and Medical Surgical Specialties, Gynecology and Obstetrics Section, University of Catania, Catania, Italy; ^5^ Department of Obstetrics and Gynecology, University Hospital of Geneva, Geneva, Switzerland; ^6^ Department of Obstetrics, Medical School, University of Crete, Heraklion, Greece

**Keywords:** ovarian stimulation, *In vitro* fertilisation (IVF)/Intracytoplasmic sperm injection (ICSI), embryo transfer, assisted reproductive techniques, ovarian response prediction

Controlled ovarian stimulation (COS) represents a crucial step in *in vitro* fertilization (IVF) cycles which aims to induce multifollicular development, with consequent retrieval of multiple oocytes.

Over the last two decades, several scientific attempts have been proposed to maximize COS efficacy. However, although well-established algorithms exist and should be used to manage women undergoing IVF, it has to be mentioned that treatments also need to be adapted to individual patients’ characteristics. In this context, tailored protocols should take into account women’s clinical history, age, and ovarian reserve.

In the current special issue, several relevant articles pave the way for new approaches in IVF treatment. Li et al. propose an easy-to-use new nomogram able to predict precisely the gonadotropin starting dose for women undergoing a GnRH antagonist protocol. A multivariate regression model based on serum anti-Müllerian hormone (AMH) level, antral follicle count (AFC), and body mass index (BMI) was developed and accounted for 59% of the variability of ovarian stimulation.

In the same vein, Liu et al. showed that patients reporting deep ovarian suppression following a GnRH agonist long protocol may benefit from a modified GnRH antagonist protocol.

Indeed, the current study describes a new strategy which suggests the administration of different dosages of cetrorelix acetate according to serum LH levels reported during COS. Results showed that ongoing pregnancy rate (OPR) and live birth rate (LBR) were much higher in the modified GnRH antagonist protocol than in the GnRH agonist long protocol group [odds ratio (OR) 3.82, 95% confidence interval (CI) 1.47, 10.61, P=0.018; OR 4.33, 95% CI 1.38, 13.60, P=0.008; respectively] with a lower cancellation rate registered in the former group.

In another interesting study (Luo et al.), the association between serum LH levels and the cumulative live birth rate (CLBR) within one complete cycle was also investigated.

Findings showed that low LH levels were associated with lower CLBRs per oocyte retrieval cycle in normogonadotrophic women who underwent COS using GnRH antagonists compared to high LH levels. In this regard, a personalized pharmacogenomic approach regarding LH supplementation based on N312S (rs2293275) LHCGR gene polymorphism may significantly increase pregnancy rate compared to the traditional approach, as suggested by Ramaraju et al.

Along this line, the use of progestin-primed ovarian stimulation as a valid alternative method to stimulate patients who present asynchronous follicular development on the fifth day of ovarian stimulation in comparison to the GnRH antagonist protocol is a novel approach that merits consideration. In particular, Dong et al. showed that a progestin-primed ovarian stimulation protocol resulted in comparable pregnancy outcomes compared to those with the standard fixed antagonist protocol. However, the rates of embryo implantation, clinical pregnancy, and early abortion were similar between the two groups, with a slightly lower number of eggs and top-quality embryos obtained in the progestin-primed ovarian stimulation group.

With regards to embryo transfer (ET), although there is evidence that progesterone levels the day of ET are crucial for HRT- FET cycles (1), a new study by Labarta et al. further adds to the available evidence by demonstrating that serum progesterone levels across luteal phase days are also associated with pregnancy outcome. Indeed, significantly higher levels of serum progesterone were observed on ET+4 (13.6 ± 6.0 vs. 11.1 ± 4.6 ng/ml, p = 0.03) and ET+11 (15.7 ± 1.2 vs. 10.3 ± 0.6 ng/ml, respectively; p = 0.000) in ongoing pregnancies versus negative β-human chorionic gonadotrophin (β-hCG) cases. Hence, serum progesterone might be playing a crucial role not only during implantation, but also in pregnancy maintenance.

In our special issue, available protocols for FET preparation are extensively presented and reviewed (Mumusoglu et al.). Except from common HRT and natural cycle (NC) or modified-NC FET protocols, the mild-OS FET protocol may also have a role. Low quality evidence shows that the NC and modified-NC protocols could be superior to HRT. On the other hand, some studies described an alarming early pregnancy loss rate in HRT cycles, with higher risk of hypertensive disorders in pregnancy obtained in cycles without a corpus luteum. Regarding warmed blastocyst transfer and timing, evidence suggests that the 6th day of starting progesterone, LH surge+6 day, and hCG+7 day in HRT, NC, and modified-NC are best, respectively. However, time corrections could be applied based on the inter-personal differences in the window of implantation or day of vitrification (day 5 or 6).

Finally, women of reproductive age are suggested to consume folic acid and other supplements before conception [also in preparation to assisted reproductive technology (ART) and during pregnancy]. The association between serum folate, magnesium (Mg), and calcium (Ca) levels before ovarian stimulation with the outcomes of ART in normogonadotropic women was recently investigated, showing that baseline elevated serum folate level (≥33.0 ng/ml) and a lower Ca/Mg ratio were associated with worse ART outcomes (Polzikov et al.).

In conclusion, from the birth of ART, COS has always been considered a crucial step to achieve successful reproductive outcomes; thus, it has been the object of continuous investigations and innovations. Although several and important goals have already been reached during the last decade, other new approaches have emerged in order to validate different treatment options which aim to treat every patient with a personalized stimulation strategy, based on an individual’s characteristics.

## Author Contributions

FDG wrote the first draft of the manuscript. NP, AM and PD were responsible for the revision process. All authors contributed to the article and approved the submitted version.

## Conflict of Interest

The authors declare that the research was conducted in the absence of any commercial or financial relationships that could be construed as a potential conflict of interest.

## Publisher’s Note

All claims expressed in this article are solely those of the authors and do not necessarily represent those of their affiliated organizations, or those of the publisher, the editors and the reviewers. Any product that may be evaluated in this article, or claim that may be made by its manufacturer, is not guaranteed or endorsed by the publisher.
